# Editorial: (Mis)perceptions of inequality as a social issue

**DOI:** 10.3389/fsoc.2026.1832125

**Published:** 2026-04-23

**Authors:** Nevena Kulic, Debora Mantovani, Daniela Bellani

**Affiliations:** 1 Department of Political and Social Sciences, University of Pavia, Pavia, Italy; 2 Department of Political and Social Sciences, University of Bologna, Bologna, Italy; 3 Department of Statistical Sciences, Catholic University of Sacred Heart, Milan, Italy

**Keywords:** attitudes, income distribution, inequality, inter-disciplinary, perceptions, subjective beliefs, wellbeing, misperceptions

Rising economic and social inequalities have become a central concern in contemporary societies (Milanovic, 2016; Piketty, 2014). Yet individuals do not often respond to inequalities as they objectively exist, but rather as they are perceived (Gimpelson and Treisman, 2018). Understanding how people perceive inequality and how these perceptions diverge from reality is therefore crucial for explaining attitudes toward redistribution, social policy, and social change (Knell and Stix, 2020; Cruces et al., 2013). This Research Topic brings together interdisciplinary contributions from sociology, psychology, political science, and media studies to explore the origins and consequences of these perceptions and misperceptions. Although the Research Topic adopts a broad understanding of inequality, encompassing both horizontal inequalities (group-based disparities) and vertical inequalities (the distribution of income and wealth), most articles have focused specifically on perceptions of economic inequality, a domain that has also received significant scholarly attention in the past (Gimpelson and Treisman, 2018).

The 11 articles included in this Research Topic span multiple traditions and methodological approaches, drawing on survey data, experimental designs, and qualitative analyses across diverse national contexts including Europe, South America, and North America. Together, they provide new insights into three interrelated questions: Do individuals misperceive inequality at societal level and their own position in the socio-economic hierarchy? What factors shape these perceptions? And how do such perceptions influence attitudes, wellbeing, and decision-making?

## Are there misperceptions of inequality? What factors influence perceptions of inequality?

Kulic, Griaznova, Clerici, Bellani et al. contribute to the Research Topic by presenting the potential of, and initial findings from, the IneqPer data, a large-scale study conducted in Italy between late 2024 and early 2025 with 12,000 respondents aged 18–70. The survey measures perceptions of inequality not only in terms of income but also across multiple domains, and incorporates several embedded experiments designed to identify the causal mechanisms underlying beliefs and preferences related to inequality. Although the findings suggest that perceptions often diverge from reality, they also show that Italians are conscious of inequalities across various dimensions.

Remaining six over 11 contributions address the presence and correlates of misperceptions. Some focus on individuals' assessments of their own position in the income distribution while others examine perceptions of broader patterns and state of societal inequality.

Two articles tackle the so-called position bias, according to which individuals tend to misperceive their relative position on the social ladder, often displaying a “center bias.” Kulic, Griaznova, Clerici confirm that individuals at the top of the income distribution are more likely to underestimate their actual position, while those at the bottom tend to overestimate theirs, a pattern that emerges both at the national and global levels of income distribution. Their analysis relies on 12,000 observations from the IneqPer survey on inequality perceptions in Italy. Similarly, Wiesner and Iturra-Sanhueza use comparative ISSP 2019 survey data from 29 countries to show a positive association between country-level income inequality and position bias, although this relationship varies across income groups. They find that low-income individuals tend to assess their position more accurately in contexts of high inequality, whereas high-income individuals are more likely to underestimate their relative standing.

The remaining four contributions explore factors that shape general perceptions of inequality patterns. These studies consider the role of beliefs, objective and subjective self-assessments, exposure to media, and the influence of meso-level contexts in which individuals are embedded.

John and Bless examine how the “belief in a just world” shapes perceptions of financial inequality by functioning as a cognitive filter. Drawing on a preregistered experiment, two representative German surveys, and a cross-national dataset spanning 40 countries, they show that stronger just-world and meritocratic beliefs are associated with lower perceived levels of inequality, both in terms of its estimated magnitude and its evaluative interpretation. Melli and Azzolini investigate how two dimensions of social position, objective social class (e.g., occupation) and subjective social status (how individuals perceive their own standing in society), influence perceptions of social inequality. Using data from the International Social Survey Programme (ISSP) covering 35 countries from 1992 to 2019, they find that perceptions of inequality are shaped more strongly by subjective social status than by objective class position. Corsbie-Massay et al. explore how audiences respond to anti-classist satire using real articles from the U.S. satirical news outlet The Onion. Based on an online study of 399 U.S. adults recruited through Prolific, a data collection platform, the findings indicate that greater appreciation of satire is linked to lower levels of agreement with beliefs that legitimize income inequality and greater confidence in discussing and challenging class inequality. Finally, Galeano-Salgado and Álvarez-Rivadulla focus on meso-level contextual factors by examining how socioeconomic diversity within universities influences students' beliefs about inequality. Drawing on 61 in depth interviews in Columbia, their findings indicate that exposure to a more socioeconomically diverse student body influences perceptions of inequality by making them more accurate, although this varies depending on students' backgrounds.

## How do perceptions of inequality shape attitudes and decisions?

Different articles examine how perceptions (and misperceptions) of inequality associate and shape individuals' attitudes, decision-making processes, and wellbeing.

Castillo et al. focus on Chile, a country characterized by profound economic and social inequalities. Using data from the Chilean Longitudinal Social Survey, they show that higher perceptions of economic inequality are associated with lower support for “market justice,” defined as the belief that higher-income individuals deserve better social services. Fernandez-Urbano examines how perceptions of macro-level labor market opportunities affect happiness through harmonized natural field experiments conducted in Pennsylvania (United States) and Barcelona (Spain). The findings reveal notable contextual differences: in Barcelona, both positive and negative perceptions influence happiness, whereas in Pennsylvania only negative perceptions affect happiness, and they do so in a detrimental way. Gilgen and Zangger introduce an innovative distributional survey experiment embedded in the 2019 Swiss MOSAICH survey to investigate how individuals balance merit, need, and equality when allocating limited resources. Their results show that individuals who perceive existing income inequality as excessive tend to distribute resources more equally.

The final contribution moves beyond economic inequality. Using data from the 2017 European Values Study across seven European countries, Guglielmi employs structural equation modeling to analyze how perceived national identity interacts with perceived threats and trust. The results show that ethnic-majoritarian conceptions of national identity are a strong predictor of support for prioritizing natives in job competition.

Across the contributions, several consistent patterns emerge. First, misperceptions of inequality appear widespread across countries and social contexts. Second, perceptions are shaped by a combination of psychological beliefs, subjective social status, and contextual environments, including exposure to diverse social settings and media narratives. Third, these perceptions have meaningful consequences for attitudes toward distributive justice, support for redistribution, and subjective wellbeing. However, the intensity and nature of these effects are context-dependent, varying across countries. Because most of the existing research focuses on perceptions of economic inequality, and the Research Topic included relatively few contributions on group-based inequality, we argue that exploring perceptions of other forms of inequality remains an important direction for future research.
